# Combined prenatal exposure to airborne polycyclic aromatic hydrocarbons and maternal distress is associated with childhood irritability

**DOI:** 10.3389/fpubh.2026.1737011

**Published:** 2026-06-02

**Authors:** Mariah DeSerisy, Daryn Wong, Huiyu Yang, Jacob W. Cohen, David Pagliaccio, Julie B. Herbstman, Virginia Rauh, Frederica P. Perera, Amy E. Margolis

**Affiliations:** 1 Department of Psychiatry and Behavioral Health, The Ohio State University, Columbus, OH, United States; 2 Department of Psychiatry, Vagelos College of Physicians and Surgeons, Columbia University, New York, NY, United States; 3 Barnard College, Columbia University, New York, NY, United States; 4 Division of Child and Adolescent Psychiatry, New York State Psychiatric Institute, New York, NY, United States; 5 Department of Environmental Health Sciences, Mailman School of Public Health, Columbia University, New York, NY, United States; 6 Columbia Center for Children's Environmental Health, Mailman School of Public Health, Columbia University, New York, NY, United States; 7 Heilbrunn Department of Population and Family Health, Mailman School of Public Health, Columbia University, New York, NY, United States; 8 The Child Mind Institute, New York, NY, United States

**Keywords:** environmental neurotoxicants, irritability, maternal psychological distress, neurodevelopment, polycyclic aromatic hydrocarbons, prenatal development

## Abstract

**Background:**

Childhood irritability is a transdiagnostic risk marker of neurodevelopmental problems. Polycyclic aromatic hydrocarbons (PAHs) and psychosocial stressors have been independently associated with altered emotional and behavioral symptoms akin to irritability, but the joint effects of PAHs and maternal psychological distress on trajectories of irritability symptoms have not been directly examined. We hypothesized that combined exposure to prenatal PAHs and maternal psychological distress would be associated with higher and more persistent irritability across childhood.

**Methods:**

Prenatal PAH was measured via personal air monitoring, and maternal psychological distress was measured via the Psychiatric Epidemiology Research Instrument–Demoralization, both measured during mothers' third trimester. Children's irritability symptoms were measured via a novel composite of item-level responses from the Children's Behavior Checklist and Conner's Parent Report Scale at ages 7, 9, and 11. Psychometric robustness of the irritability composite scores (N = 511) were evaluated. Latent growth curve modeling (LGM) examined interactive effects of PAH and maternal distress on irritability trajectories (N = 394 with complete exposure and outcome data). Follow-up linear regression analyses tested associations at ages 9 and 11.

**Results:**

The irritability composite score was unitary and demonstrated good psychometric properties. Combined exposure to prenatal PAH and maternal distress exposure was associated with higher irritability at ages 7, 9 and 11, but not with change in irritability from age 7–11.

**Conclusions:**

The novel irritability composite had a unitary factor structure reflecting a single irritability construct. Combined prenatal exposure to PAHs and maternal psychological distress was associated with higher irritability scores at every developmental timepoint suggesting that chemical exposures may exacerbate the effects of maternal distress on child irritability or vice versa. Exposure to either was not associated with change in irritability over time.

## 1 Introduction

Early life adversity can have long-lasting detrimental effects on human health via increased risk for stress-related disease (1, 2). Adverse exposures can occur through environmental exposure to chemicals, such as those found in air pollution, or through psychosocial stressors, such as family dysfunction or trauma (3–6). Critically, the timing of exposure can influence the magnitude of long-term effects, with the prenatal period being especially vulnerable because it is a period of rapid brain development (7, 8). Increasing evidence suggests that chemical exposures and social stressors act on similar neurobiological stress pathways and thus may act synergistically to alter children's neurodevelopment (9).

One transdiagnostic marker of psychiatric and neurodevelopmental risk is irritability (10–13). It often reflects heightened physiological reactivity to psychosocial stressors coupled with a developmentally inappropriate ability to regulate one's behavioral and emotional responses to those stressors (14, 15). In clinical samples, childhood irritability is characterized by consistently angry/grumpy mood, and/or temper outbursts (physical or verbal aggression, crying, or whining) and increases risk for future psychiatric concerns (16). In nonclinical samples, higher irritability is also linked to greater functional impairment (17). Thus, it is important to identify early-life risk factors for irritability (18–21). Critically, limited research suggests that irritability development may be different among those from different ethnoracial backgrounds (22, 23), highlighting the need for additional research in diverse samples.

Polycyclic aromatic hydrocarbons (PAHs) represent one class of chemical neurotoxicants that can alter children's neurodevelopment (24). PAHs are ubiquitous air pollutants produced by the incomplete combustion of organic materials such as fossil fuels and tobacco smoke (25, 26). Animal models indicate that PAHs can cross the placental barrier and influence fetal brain development via oxidative stress and inflammation to result in long-term, cascading effects on offspring (27, 28). In humans, prenatal PAH exposure is associated with increased risk for neurodevelopmental problems in children, including those characteristic of irritability, such as emotional instability, hyperactivity, and angry or aggressive behaviors (13, 24, 29–32). Additionally, prenatal PAH exposure is associated with increased symptoms of anxiety and depression, common adolescent endpoints for persistent childhood irritability (33), though these findings are less robust (24, 34, 35). Thus, prenatal PAH exposure may alter the course of children's brain development in ways that increase risk for irritability; however, limited research has tested these associations.

Psychosocial stressors, including those associated with living in disadvantaged contexts, parental mental health problems (e.g., depression), harsh parenting, exposure to trauma and community violence, and family dysfunction, have consistently been linked to elevated irritability and emotion dysregulation (22, 35–45). In particular, maternal psychological distress has been associated with childhood irritability through both genetic (46) and environmental pathways. Additionally, maternal psychological distress can disrupt early attachment and sensitive caregiving, potentially altering the course of children's emotional development (47–49). Further, associations between children's brain development and maternal psychological distress are seated in stress-response and neuroendocrine pathways (7, 50), which are the same biological pathways targeted by PAHs (51–54). Critically, exposure to PAHs is most common among families at greatest risk of exposure to psychosocial stressors (55–57), pointing to a “two-hit” framework that combined prenatal exposure to PAHs and maternal psychological distress may increase risk for clinically significant irritability (9).

Herein, we study the interacting effects of prenatal maternal psychological distress and PAH exposure on trajectories of childhood irritability (ages 7–11) in a prospective longitudinal birth cohort (N = 394). We examine exposure in the prenatal period because it is a vulnerable time in child development (7, 58, 59). We hypothesized that combined exposure to PAH and maternal psychological distress during pregnancy would be associated with greater irritability at age 7 and more persistent irritability across childhood.

## 2 Materials and methods

### 2.1 Participants

Data used in the current study was collected as a part of an ongoing longitudinal prospective birth cohort study conducted at the Columbia Center for Children's Environmental Health (CCCEH); detailed demographics and recruitment information have been previously published (60). Briefly, participants were Black and Latiné women recruited between 1998 and 2006 through prenatal care clinics in Northern Manhattan and Washington Heights in New York City. Recruited women (ages 18–35 years) were non-users of tobacco products or other substances, and were free of diabetes, hypertension, or known HIV, and had initiated prenatal care by the 20th week of pregnancy. A total of 727 mother-child dyads were included in the cohort. Development of the childhood irritability composite included all cases with any available irritability data (N = 511; Table 1). Of the 727 dyads enrolled, 394 had available data for all predictors of interest (i.e., PAHs, maternal psychological distress, irritability at any timepoint) and so were included in the latent growth curve analyses; cases with missing data were deleted listwise. The cohort study is approved by the Institutional Review Board of Columbia University; mothers provide informed consent for themselves and their children at every study visit. Children provide assent beginning at age 7.

**Table 1:** Irritability item level data available at each age point.

Test	Age (years)	N
Child behavior checklist	7	511
	9	462
	11	367
Conners parent rating scales	7	504
	9	467
	11	366

### 2.2 Study timeline

Longitudinal study visits began in the third trimester of pregnancy and occurred approximately every 2 years thereafter. The current study leverages data collected during four visits: “prenatal” (approximately mother's third trimester of pregnancy, range: 18.71–48.00 gestational weeks; Mean gestational weeks = 33.22), child “age 7” (range: 6–8 years; mean age = 6.52 years), “age 9” (range: 8–10 years; mean age = 8.51 years), and “age 11” (range: 10–13 years; mean age = 10.56 years). Parent report measures of children's irritability were collected during children's age 7, 9, and 11 year visits; maternal psychological distress (self-report) and PAH exposure (personal air monitoring) were measured during the mother's third trimester of pregnancy.

### 2.3 Measures

#### 2.3.1 Maternal psychological distress

We measure one aspect of maternal psychological distress, maternal demoralization, using the Psychiatric Epidemiology Research Instrument–Demoralization [PERI-D; (61)]. The PERI-D is a 27-item scale measuring eight composite domains of non-specific psychological distress: perceived physical health, sadness, poor self-esteem, dread, anxiety, confused thinking, hopelessness/helplessness, and psychophysiological symptoms. Our prior work showed that higher PAH exposure during pregnancy was associated with higher maternal psychological distress concurrently and worse mental health symptoms for their children in adolescence (62). We now test if the interaction between prenatal PAH and maternal psychological distress exposure is associated with a worse trajectory of irritability symptoms (33). Average PERI-D score was mean centered and scaled for all analyses; participants with Z-score values above or below 3 were considered outliers and winsorized to the next non-outlier value.

#### 2.3.2 PAH exposure

Full details regarding the acquisition and analysis of maternal air pollution exposure data have been described previously (57, 60). Briefly, mothers wore a backpack containing a personal air monitor during daytime hours (i.e., all waking hours) for two consecutive days; the monitor was placed next to the bed at night. The personal air sampling pumps operated continuously, collecting vapors and particles of ≤ 2.5 μg in diameter on a precleaned quartz microfiber filter and a precleaned polyurethane foam cartridge backup. Southwest Research Institute analyzed samples for eight carcinogenic PAHs. Herein, PAH exposure was operationalized as the natural logarithm of the sum of the 8 available PAHs over the entire collection period (57, 60, 63, 64). Measured PAHs included: Benz (a) anthracene, Benzo (a) pyrene, Benzo (b) fluoranthene, Benzo (g, h, i) perylene, Benzo (k) fluoranthene, Chrysene, Disbenz (a, h) anthracene, Indeno (1,2,3-cd)pyrene (57, 60, 63, 64). Details of quality control of air monitoring assessment have been previously reported (63, 64), and can be found in the supplement. Participants with Z-score values above or below 3 were winsorized to the next non-outlier value.

#### 2.3.3 Childhood irritability

Parent rating of child irritability in children ages 7–11 was quantified using item-level response data (Table 2) across two questionnaires [Children's Behavior Checklist [CBCL; (65)] and Conners Parent Report Scale; (CPRS; (66))]. We use 3 items from the CBCL that have been used as markers of irritability by other researchers in the field [(67, 68); irritable mood, sudden changes in mood, and hot temper] and 7 items from the CPRS that capture aspects of irritability, including items such as temper outbursts that are not captured on the CBCL. The exact wording of each item is presented in Table 2.

**Table 2:** Items extracted from individual scales.

Measure	Relevant item(s)
Child behavior checklist	86. irritable mood
	87. sudden changes in mood
	95. hot temper
Conners parent rating scales	1. Angry and resentful
	21. Loses temper
	31. Irritable
	47. Temper outbursts
	68. Demands must be met immediately – easily frustrated
	77. Mood changes quickly and drastically
	78. Easily frustrated in efforts

Item-level data across scales were reverse-coded as necessary and Z-scored to ensure that all data were interpretable on the same scale, with higher scores indicating more irritability. Responses were averaged within each of the scales, and then averaged across parent-report scales to create a single composite score (range = −1 to 3), reflecting parent-report of child irritability at each age.

To validate the irritability composite, fit indices from exploratory factor analysis, internal consistency, test-retest reliability, and construct validity were examined using all available data (N = 511). Exploratory factor analysis model fit was assessed using root mean square error of approximation (RMSEA), comparative fit index (CFI), and the standardized root mean square residual (SRMR) as these are recommended for smaller sample sizes [N < 500; (69, 70)]. To indicate a valid scale, the RMSEA should be close to zero with a significance value > 0.05 (71, 72), CFI > 0.90 (72, 73), and SRMR < 0.08 (74). Because items from two scales were combined, we explored single, two factor, and bifactor solutions at each age point. According to Finch and colleagues (75), to be considered a significantly better model, CFI must improve by > 0.01, RMSEA must improve by > 0.015, and the SRMR must improve by > 0.03. Cronbach's alpha was used to assess internal consistency of the optimal factor solution. Test-retest reliability was investigated via Pearson's correlations and intraclass correlations (ICC) at all three timepoints with ICC interpreted as: between 0.5–0.75 moderate, and 0.75–0.9 good (76). Construct validity was assessed via convergent validity, established when a new measure demonstrates a predicted correlational pattern with established measures (77, 78). We predicted positive correlation of the new irritability composite with the Lability/Negativity Index and inverse correlation with the Emotion Regulation Index of the parent-report form of the Emotion Regulation Checklist (79) in a subset of children at age 9. To assess robustness of findings, supplementary analyses separately examined composite scores using the CBCL or CPRS items. After scale development, scores were mean-centered and scaled; outliers |Z = ±3| were winsorized. Winsorized, scaled scores were used in all analyses.

### 2.4 Statistical methods

All variables were mean-centered and scaled prior to inclusion in analyses (80, 81). As described above, outliers in all exposures and outcomes of interest were winsorized to the next non-outlier value (Supplementary Table S1 for details). Chi-square or independent samples *t*-tests were used to assess for differences between included and excluded participants. Correlations among untransformed variables were examined; correlations between variables *r* > 0.7 are considered problematic (82). Missing data were examined for demographic differences between those included and excluded in this current study (Supplementary Table S2) and for non-random patterns of attrition (see Supplemental results).

Next, we tested our main hypothesis that greater exposure to PAH and maternal psychological distress is associated with higher irritability in children using a latent growth curve model (lavaan (83); R studio version 2025.09.1). Of note, latent growth curve models are distinct from latent class models in that they use the continuous effects of all predictors on the continuous, data-derived outcomes, namely intercept and slope scores. Intervals between slope loadings were scaled to reflect the average interval (i.e., years) between study visits. Model fit was assessed using RMSEA, CFI, and SRMR, as described above (69, 70). In the case that the association between exposure and the intercept or slope was not estimable and nonsignificant, we planned to conduct follow-up linear regressions analyses. For the intercept, we planned to examine associations between the exposure and irritability at each timepoint. For the slope, we planned to examine associations between exposures and irritability at each later time point (i.e., 9 and 11). All cases with any available irritability data were used (N = 394). The latent growth curve model flexibly handled sample size disparities by including all available cases in the model at each step; missing data was handled using full-information maximum likelihood. Sensitivity analyses were conducted in the sample with complete irritability data for all waves. Sensitivity analyses were additionally conducted in the subsample of participants with scaled scores |Z| < 3 for all exposures and outcomes (i.e., outliers were removed rather than winsorized). The RMSEA should be close to zero with a significance value > 0.05 (71, 72), CFI > 0.90 (72, 73), and SRMR < 0.08 (74). A subgroup analysis was conducted in mothers with the highest quartile prenatal demoralization scores. To test the robustness of our findings, sensitivity analyses were additionally conducted using only items from the CBCL or CPRS.

All models were adjusted for hypothesized confounders or variables that improved the precision of the estimates including child sex at birth (84, 85), maternal years of education as a proxy for socioeconomic status (86, 87), maternal age at child's birth (88, 89), quality of the home environment (90), maternal intelligence ((91–93); Test of Nonverbal Intelligence [TONI; (94)]), child birth weight (95, 96), maternal ethnoracial identification (24, 63), maternal nativity (97), the presence of a smoker in the home (98, 99), heating season when PAH was measured (100–102), and a dichotomous variable identifying if the family had moved between the prenatal and age 5 visits to account for changes in pollution exposure across childhood (103–105); See Supplementary methods for details about the assessment of covariates.

## 3 Results

### 3.1 Participants

Table 3 presents demographic information of the sample included in the main analyses. Results of attrition analyses are available in the supplement. Black mothers were more likely to be excluded from the current sample for missing data than Latiné mothers (Supplementary Table S2). Detailed demographics of participants included longitudinally can be found in Supplementary Table S3.

**Table 3:** Demographic characteristics.

	Total
N	394
Mean child age in years (SD)	7.5 (2.34)
Sex (% male)	272 (49.46)
Average prenatal PAH exposure (SD)^a^	0.84 (0.73)
Average maternal demoralization (SD)	0.42 (0.13)
Smoker at home (% yes)	185 (33.27)
Heat season (% yes)	275 (49.19)
Child birthweight in grams (SD)	3370 (474.49)
Maternal nativity (% Native)	270 (48.21)
Maternal age at child birth (SD)	24.81 (4.91)
Maternal years of education at prenatal visit (SD)	11.89 (2.16)
Maternal IQ (SD)	85.09 (13.16)
Average home environment score (SD)^b^	39.28 (6.37)
Moved (% yes)	146 (29.67)
Latiné (%)	352 (62.74)
Black (%)	209 (37.25)

^a^Z-scaled natural logarithm of prenatal PAH exposure measured by personal air monitoring in the third trimester.

^b^ Quality of the home environment measured by total score from HOME scale.

N, number of participants included in the main analytic model; SD, standard deviation; PAH, polycyclic aromatic hydrocarbons; IQ, Intelligence Quotient.

### 3.2 Assessment of irritability composite

**Figure 1:**
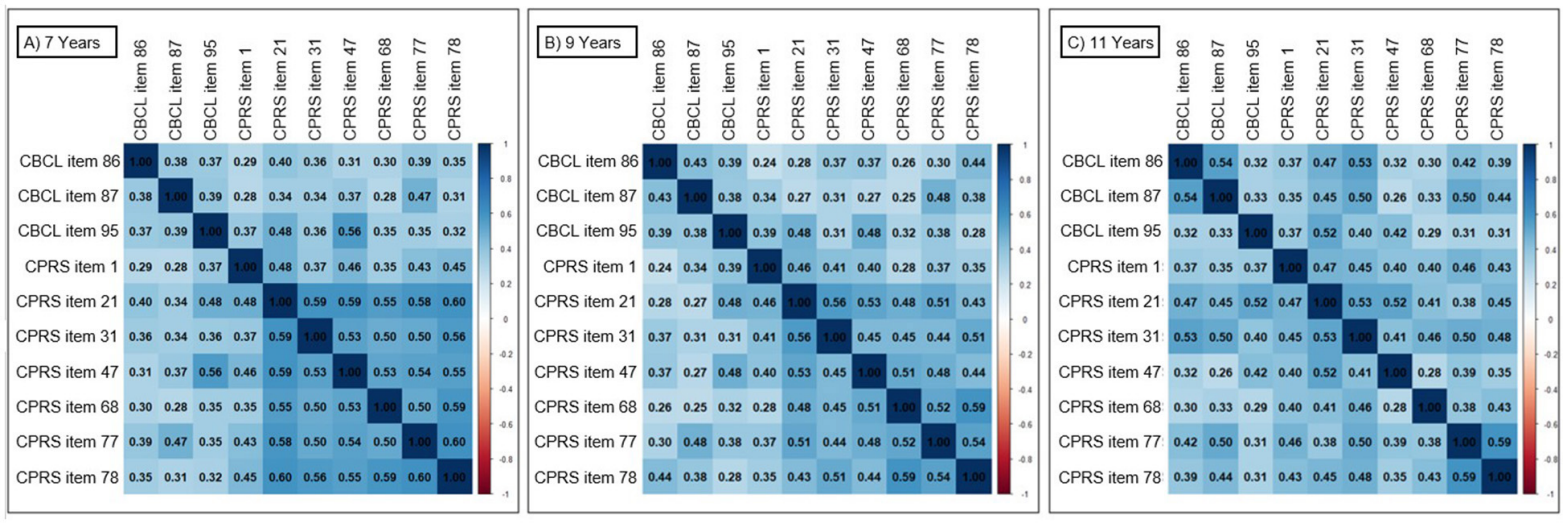
Irritability Intra-Item Correlations at **(A)** Age 7 Years, **(B)** Age 9 years, and **(C)** Age 11 Years. Scale color denotes strength of correlations, blue = positive, red = negative. CBCL, Child Behavior Checklist, CPRS, Conners Parent Rating Scale.

**Figure 2:**
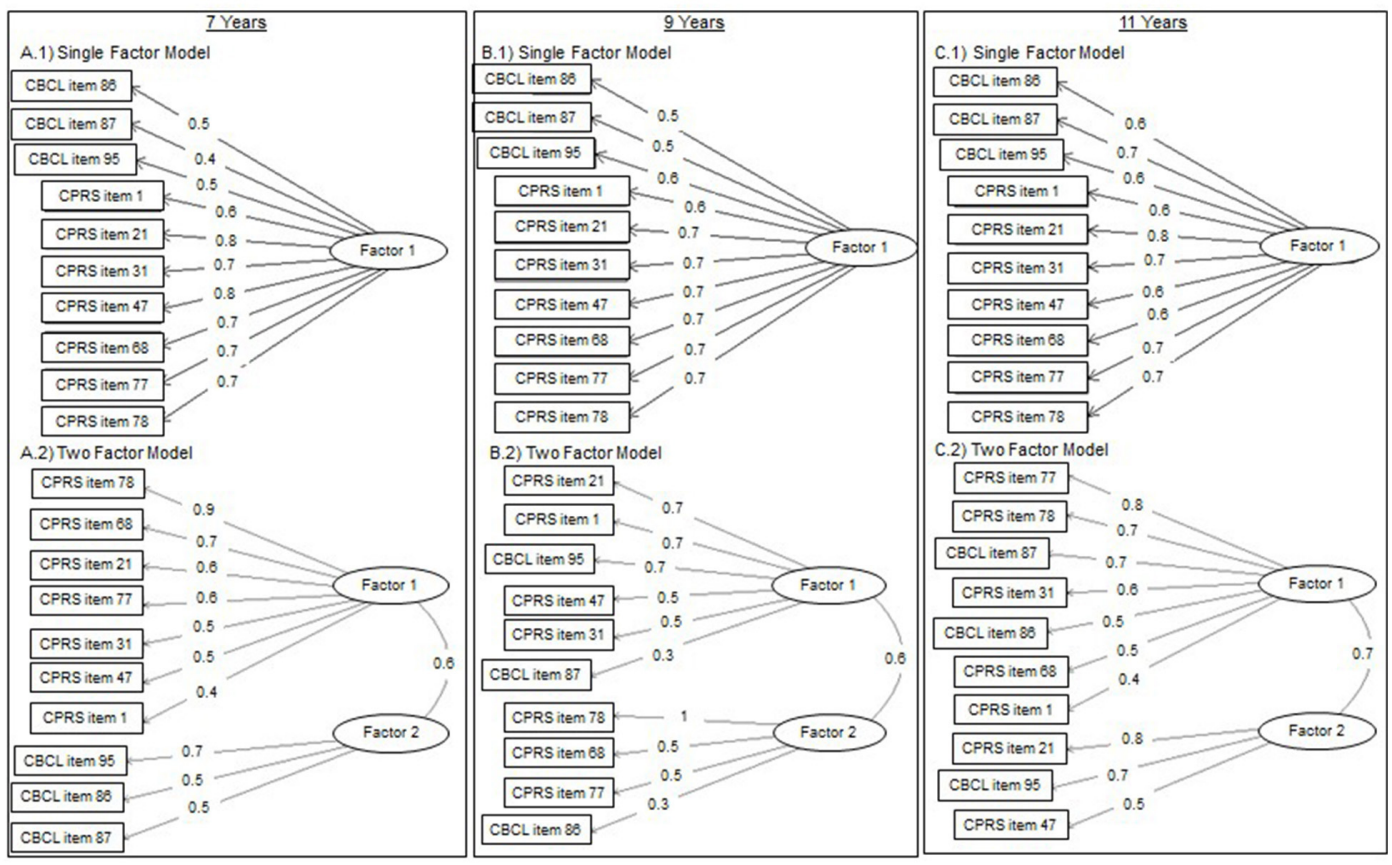
Factor loadings of irritability items at **(A)** Age 7 Years, **(B)** Age 9 years, and **(C)** Age 11 Years. CBCL = Child Behavior Checklist, CPRS = Conners Parent Rating Scale.

**Figure 3:**
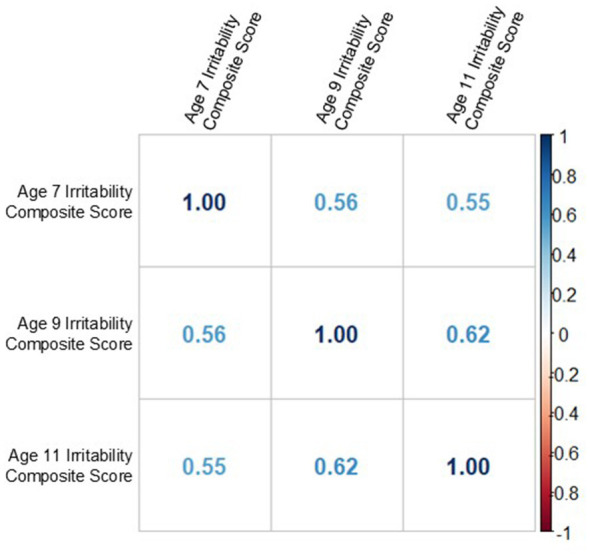
Correlations between irritability composite scores across age points.

Figure 1 shows item correlations across all time points. Exploratory factor analysis identified a single factor solution at age 7 and 11 (Table 4) as the two-factor solution indicated only marginal improvement in fit [Table 4; Figure 2; (75)]. Bifactor analyses did not converge. Internal consistency of single factor irritability composite scores were α = 0.884, 0.870, and 0.874 at each timepoint, respectively. Irritability composite scores were significantly inter-correlated (Figure 3). Across all three time points, single measure ICC was 0.579 [95% CI (0.522, 0.634), *p* < .001) and average-measure ICC was 0.805 [95% CI (0.766, 0.838), *p* < .001]. In support of construct validity, the age 9 irritability score was positively correlated with parent report lability/negativity (*r* = 0.55, *p* > 0.001) and negatively correlated with parent report emotion regulation (*r* = −0.12, *p* = 0.017).

**Table 4:** Model fit indices for one- and two-factor exploratory factor analyses.

Model	CFI	RMSEA	RMSEA CI lower bound	RMSEA CI upper bound	SRMR
Age 7					
Single–factor	0.924	0.090	0.071	0.110	0.054
Two–factor	0.945	0.078	0.058	0.099	0.046
Age 9					
Single–factor	0.859	0.125	0.107	0.144	0.068
Two–factor	0.877	0.118	0.099	0.137	0.064
Age 11					
Single–factor	0.889	0.111	0.093	0.131	0.059
Two–factor	0.903	0.105	0.086	0.125	0.059

CFI, comparative fit index; RMSEA, root mean square error of approximation; CI, confidence interval; SRMR, standardized root mean square residual.

### 3.3 Intercorrelations between variables included in main analyses

Intercorrelations between untransformed confounders were all below 0.32, indicating no problems with collinearity [(75); Supplementary Figure S1].

### 3.4 Combined exposure to PAH and maternal psychological distress is associated with irritability

The latent growth model of the interaction between prenatal PAHs and maternal PERI-D scores on the irritability intercept and slope indicated an excellent model fit (RMSEA = 0.019, RMSEA *p*-value = 0.928, CFI = 0.993, SRMR = 0.010; Table 5). When not accounting for exposures, neither the baseline irritability score (intercept) nor the average irritability change over time (slope) were different from zero (β_*intercept*_ = 0.066; *p*_*intercept*_= 0.696; β_*intercept*_ = −0.027; *p*_*slope*_= 0.560). The covariance of the intercept and the slope was also not significantly different from 0 (β = 0.031; *p* = 0.185), indicating that child irritability scores at age 7 do not correlate with change in child irritability scores over time when not accounting for exposures.

**Table 5:** Latent growth curve model results.

Variable	Intercept					Slope				
	B	Beta	SE	*p*–value	Confidence interval	B	Beta^*^	SE	*p*–value	Confidence interval
Prenatal PAH exposure^a^	−0.327	−0.488	0.150	0.029	(−0.621, −0.034)	0.034	–	0.044	0.450	(−0.054, 0.121)
Maternal demoralization	0.152	0.228	0.049	0.002	(0.057, 0.248)	0.010	–	0.014	0.475	(−0.054, 0.121)
Interaction (PAH X MD)	0.870	0.557	0.346	0.012	(0.193, 1.547)	−0.067	–	0.099	0.498	(−0.261, 0.127)
Sex	0.243	0.187	0.091	0.007	(0.065, 0.422)	−0.049	–	0.025	0.055	(−0.099, 0.001)
Birth weight	0.009	0.014	0.045	0.843	(−0.080, 0.098)	−0.003	–	0.013	0.781	(−0.028, 0.021)
Race	0.018	0.028	0.068	0.790	(−0.115, 0.151)	0.014	–	0.019	0.444	(−0.023, 0.051)
Smoker at home	0.042	0.065	0.049	0.395	(−0.054, 0.138)	−0.025	–	0.014	0.062	(−0.052, 0.001)
Maternal IQ	−0.032	−0.050	0.050	0.525	(−0.130, 0.066)	0.036	–	0.014	0.011	(0.008, 0.063)
Maternal years of education	0.087	0.128	0.054	0.107	(−0.019, 0.194)	−0.006	–	0.016	0.711	(−0.037, 0.025)
Home environment^b^	−0.057	−0.085	0.051	0.264	(−0.156, 0.043)	0.010	–	0.015	0.513	(−0.019, 0.038)
Moved	−0.001	−0.002	0.046	0.978	(−0.091, 0.088)	0.006	–	0.013	0.623	(−0.019, 0.031)
DOB heat season	−0.089	−0.068	0.095	0.350	(−0.275, 0.097)	0.020	–	0.026	0.445	(−0.031, 0.072)
Maternal nativity	−0.022	−0.017	0.145	0.881	(−0.306, 0.263)	0.028	–	0.040	0.484	(−0.050, 0.106)
Maternal age at child birth	−0.012	−0.018	0.051	0.820	(−0.111, 0.088)	0.008	–	0.014	0.578	(−0.019, 0.035)

B, unstandardized coefficient; Beta, standardized coefficient; SE, standard error; ^*^=standardized coefficients for slope predictors were unavailable due to minimal interindividual variability. ^a^ Z-scaled natural logarithm of prenatal PAH exposure measured by personal air monitoring in the third trimester. ^b^ Quality of the home environment measured by total score from HOME scale. N= number of participants; PAH, polycyclic aromatic hydrocarbons; MD, Maternal Demoralization; IQ, Intelligence Quotient; DOB, Date of birth.

**Figure 4:**
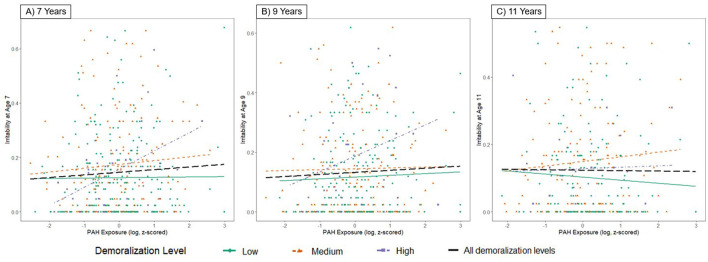
Interaction between Maternal Demoralization and PAH Exposure on **(A)** Age Seven Irritability, **(B)** Age 9 Irritability, and **(C)** Age 11 Irritability. Maternal demoralization was stratified for visualization; Purple squares and long dashed line = High Demoralization; Orange triangles and short dashed line = Mid-level Demoralization; Green circles and solid line = Low Demoralization.

The interaction between prenatal PAHs and maternal PERI-D scores was associated with the intercept of irritability scores such that higher combined prenatal exposure to PAHs and more maternal psychological distress was associated with higher irritability scores at age 7, representing a large effect (B = 0.870, β = 0.557, *p* = 0.012; Figure 4). Prenatal PAH was inversely associated with age 7 irritability (i.e., intercept; B = −0.327, β = −0.488, *p* = 0.029); maternal PERI-D scores were positively associated with higher age 7 irritability (i.e., intercept; B = 0.152, β = 0.228, *p* = 0.002). The interaction between PAH and maternal PERI-D scores did not predict the change in childhood irritability (B = −0.067, *p* = 0.498); main effects of PAH (B = 0.034, *p* = 0.450) and maternal PERI-D scores (B = 0.010, *p* = 0.475) on the slope of childhood irritability were also nonsignificant. Of note, standardized coefficients could not be calculated for slope predictors due to limited variance in change scores. The pattern of results was similar in the subgroup analysis in mothers with highest quartile prenatal demoralization (Supplementary Table S6). The pattern of results was also similar in the sample with complete irritability data, in models including only the CBCL or the CPRS, and in models with outliers removed rather than winsorized (Supplementary Tables S7-S10).

Given that the slope was not estimable and nonsignificant in the latent growth model, follow-up analyses examined associations between exposures and irritability at ages 9 and 11 (Table 6, Figure 4). The interaction between PAH and maternal PERI-D scores was associated with age 9 irritability scores, such that children exposed to higher levels of prenatal PAHs and more maternal distress demonstrated higher irritability scores (*p* = 0.013). Maternal psychological distress was positively associated with age 9 irritability (β = 0.204; *p* = 0.0002); prenatal PAH exposure was inversely associated with age 9 irritability (β = −0.375; *p* = 0.028). The interaction between PAH and maternal PERI-D scores predicted age 11 irritability scores such that children exposed to higher levels of prenatal PAHs and maternal psychological distress demonstrated higher irritability scores (*p* = 0.035). Maternal psychological distress was positively associated with age 11 irritability (β = 0.176; *p* = 0.006); PAH was not associated with age 11 irritability (β = −0.375; *p* = 0.063).

**Table 6:** Linear regression model results.

Variable	Age 9			Age 11		
	Coefficient	z-value	*p*–value	Coefficient	z–value	*p*–value
Prenatal PAH exposure^a^	−0.375	−2.210	0.028	−0.375	−1.864	0.063
Maternal demoralization	0.204	3.747	0.000	0.176	2.796	0.006
Interaction (PAH X MD)	0.971	2.510	0.013	0.946	2.121	0.035
Sex	0.209	2.081	0.038	0.064	0.550	0.583
Birth weight	0.008	0.169	0.866	−0.027	−0.468	0.640
Race	0.163	2.201	0.028	0.090	1.050	0.294
Smoker at home	−0.045	−0.827	0.409	−0.025	−0.403	0.687
Maternal IQ	0.009	0.158	0.874	0.137	2.143	0.033
Maternal Years of education	0.080	1.274	0.204	0.063	0.847	0.398
Home environment^b^	−0.049	−0.837	0.403	−0.043	−0.627	0.531
Moved	0.020	0.386	0.700	−0.021	−0.357	0.721
DOB heat season	−0.138	−1.327	0.185	0.131	1.099	0.273
Maternal nativity	−0.121	−0.760	0.448	−0.004	−0.023	0.982
Maternal age at child birth	0.021	0.376	0.707	−0.006	−0.095	0.924

^a^Z-scaled natural logarithm of prenatal PAH exposure measured by personal air monitoring in the third trimester.

^b^Quality of the home environment measured by total score from HOME scale.

N, number of participants; PAH, polycyclic aromatic hydrocarbons; MD, Maternal Demoralization; IQ, Intelligence Quotient; DOB, Date of birth.

## 4 Discussion

The current study examined if prenatal exposure to PAH air pollution combined with maternal psychological distress was associated with childhood irritability in N = 394 Black and Latiné youth living in economically disadvantaged contexts in New York City. To address this question, we first developed and assessed a novel irritability composite including face valid items from commonly used scales. Next, we show that combined exposure to prenatal PAH and maternal psychological distress was significantly associated with higher irritability at age 7, 9, and 11, but not changes in irritability from age 7 to 11. These findings suggest that children with higher exposure may have persistently higher levels of irritability and that, within this population of youth, irritability may not decline from age 7 to 11 as developmentally expected.

To study irritability in this prospective, epidemiologic cohort sample, we first derived a novel irritability composite with good psychometric properties from items included in the Child Behavior Checklist and the Conners Parent Report Scale. The three-item irritability scale derived from the Child Behavior Checklist has commonly been used in irritability research, but its psychometric robustness has been debated (67, 106, 107). As such, we combined those three items with items from the Conners Parent Report Scale to improve our estimates of irritability. Results suggest that these items can function as a single factor with good test-retest reliability, moderate stability, and acceptable internal consistency in all 3 waves in a sample of Black and Latiné urban youth. However, model fit at age 9 was less robust than at age 7 or 11. As such, future research should test the reproducibility of our findings in a large, nonclinical sample. Specifically, additional research in heterogeneous epidemiologic samples is needed to improve generalizability; however, few extant prior epidemiologic studies collected gold-standard irritability measures. Moreover, irritability measurement in nonclinical samples remains a primary concern for the field (107, 108). Thus, the development of a novel irritability composite with good psychometric properties from scales commonly administered in prospective, epidemiologic cohorts was a critical first step toward improving retrospective irritability research and sets the stage for similar analyses in other open-science datasets.

In support of a “two-hit” framework of chemical and psychosocial stress exposure, children with higher prenatal exposure to both PAH and maternal distress had higher irritability at all measured ages. This observed association could derive from the effect of combined exposure on a shared neurobiological pathway. PAHs cross the placental barrier and accumulate in fetal brain tissue, where they alter gene expression related to neurodevelopment, particularly in pathways governing synaptogenesis, dopaminergic function, and stress responsivity (109–111). Maternal psychological distress, including demoralization, is associated with elevated glucocorticoids and altered maternal immune activation, which in turn can affect fetal brain development and placental function (7, 112). In support of this hypothesis, in rodent models, combined exposure to air pollutants and maternal stress during gestation results in amplified neuroinflammatory signaling, impaired synaptic pruning, altered expression of oxytocin and vasopressin, and increased anxiety- and depression-like behaviors in offspring (113, 114). Similarly, prenatal PAH exposure in humans has been associated with emotional reactivity, attention problems, and externalizing symptoms in young children (30, 34), and these effects are exacerbated by maternal psychological distress during pregnancy (62, 115, 116). Of note, the current findings show that PAH and maternal distress contribute to persistently high irritability, rather than to changes in the developmental course of irritability over time, pointing to the possibility that residual confounding or shared method variance could also explain the findings. However, the rigorous epidemiologic design and prior findings in this cohort that mother's report of child behavior is not influenced by maternal mental health (29) support the interpretation that combined exposure to air pollution and psychosocial stress influence irritability over childhood.

We did not detect overall changes in irritability symptoms over time. This finding is in contrast to prior research showing that, although normative in preschool age children (16, 117), irritability symptoms typically decline over childhood (22, 36). The nonsignificant slope in our study may indicate that change over time in irritability in this sample differs from developmental trends reported in other samples (22, 36). In line with prior work (22, 23, 118), trajectories of irritability may operate differently in our sample of Black and Latiné urban youth than in suburban cohorts of predominantly Caucasian descent (e.g., (119)). Given that prior work has observed considerable heterogeneity in intraindividual trajectories (22, 36), it is also possible that the nonsignificant slope is related to sample size or limited power; however, similarly sized cohorts with different demographics have reported decreasing symptoms over childhood (117). Discrepancies in findings across studies may also be explained by differences in irritability measurement tools (106). It is also possible that limited variance in irritability scores in our sample constrained the rate of change and contributed to null findings. To disentangle effects attributable to measurement tools and accurately identify developmental trajectories of irritability, future studies should include varied populations and multimethod approaches to measuring irritability.

The results from this study should be considered in light of several strengths and limitations. The focus on irritability provides insight into an important and relatively understudied transdiagnostic construct that is both prevalent and predictive of multiple psychiatric outcomes (10, 120, 121). PAH exposure was measured objectively using personal air monitors worn during the third trimester, a validated, personally relevant approach used in prior studies of prenatal environmental risk (57). Further, the study leveraged repeated behavioral assessments over 4 years, allowing modeling of irritability trajectories. However, we were unable to account for residual confounding from postnatal exposures, such as continued environmental pollution, social stressors (e.g., peer conflict), neighborhood-level factors (e.g., poverty, built environment), parenting quality, or child-level risk factors (e.g., executive function deficits), which may also influence irritability persistence (10, 24, 122–124). Although the prenatal period marks a critical window of vulnerability due to rapid brain development (7), unmeasured postnatal exposures may contribute to the observed associations. We also did not measure maternal mental health symptoms separate from demoralization. Accordingly, our findings cannot disentangle effects attributable to any single diagnostic domain or differentiate between clinically significant and nonclinical elevations in maternal psychological distress. Despite this, our findings extend prior research to encompass nonclinical levels of perinatal psychological distress, which are experienced by as many as thirty percent of mothers (125, 126), and highlight an important and often overlooked marker of experience in a population that may benefit from early identification and support. Because demographic differences were detected between dyads included versus excluded from the study, we control for potential confounding by including these variables as covariates in all analyses and note that these differences could limit generalizability and introduce selection bias. Finally, irritability was assessed via parent report, which may be influenced by maternal characteristics. Specifically, shared method variance or informant bias may have contributed to results; however, it is unlikely that either fully explain the observed interaction. Limited evidence supports the hypothesis that mother's reports of their children's mental health is negatively biased by their own mental health status [for review: (127–136)]. Further, in our cohort we have previously shown that mother's report of child behavior is not influenced by maternal mental health (29).

## 5 Conclusions

Combined prenatal exposure to PAHs and maternal psychological distress was associated with higher irritability across childhood, but not change in irritability over time. These results align with a “two-hit” framework of chemical and psychosocial stress exposure such that exposure to neurotoxicant chemicals may alter the course of development, leaving children more vulnerable to effects of psychosocial stressors and downstream maladaptive neurobehavioral outcomes (9). Moreover, irritability symptoms in this sample did not decline over time, suggesting that our irritability measure may have more accurately captured differences in the developmental trajectories of irritability among Black and Latiné urban youth than has been observed in prior work. These findings underscore the importance of addressing cumulative prenatal risk exposures in an effort to reduce early behavioral vulnerability and prevent downstream mental health problems. Future work should examine the mechanistic pathways linking combined prenatal stressors to child irritability and test whether postnatal interventions, such as reducing household air pollution or providing maternal mental health support, can modify these developmental trajectories.

## Data Availability

The data analyzed in this study is subject to the following licenses/restrictions: Data is available upon written request. Requests to access these datasets should be directed to Julie Herbstman; jh2678@cumc.columbia.edu.
