# Supplementary material for: Combined prenatal exposure to airborne polycyclic aromatic hydrocarbons and maternal distress is associated with childhood irritability
Source: Front Public Health. 2026 Jun 2;14:1737011. doi: 10.3389/fpubh.2026.1737011 (PMC13269327; doi:10.3389/fpubh.2026.1737011)
Supplement: Supplementary file 1 [file Data_Sheet_1.docx]

Supplement to Combined Prenatal Exposure to Airborne Polycyclic Aromatic Hydrocarbons and Maternal Distress is Associated with Childhood Irritability

Mariah DeSerisy, PhD;^a,b*^ Daryn, Wong,^c^ Huiyu Yang, M.Sc.^a,d^, Jacob W Cohen, BA;^b,d^ David Pagliaccio, PhD;^a,d^ Julie Herbstman, PhD;^e,f^ Virginia Rauh, ScD, MSW.;^f,g^ Frederica P. Perera, DrPH, PhD;^e,f^ Amy E. Margolis, PhD^a,h^

1. Department of Psychiatry and Behavioral Health, The Ohio State University, Columbus, OH 43210
2. Department of Psychiatry, Vagelos College of Physicians and Surgeons, Columbia University, New York, NY, 10032
3. Barnard College, Columbia University, New York, NY 10027
4. Division of Child and Adolescent Psychiatry, New York State Psychiatric Institute, New York, NY, 10032
5. Department of Environmental Health Sciences, Mailman School of Public Health, Columbia University, New York, NY 10032
6. Columbia Center for Children’s Environmental Health, Mailman School of Public Health, Columbia University, New York, NY 10032
7. Heilbrunn Department of Population and Family Health, Mailman School of Public Health, Columbia University, New York, NY 10032
8. The Child Mind Institute, New York, NY, 10022

Short title: Prenatal Exposures Impact Child Irritability

Supplementary Methods

Personal Air Monitoring Quality Control Assessment.

As described in prior work (1,2), to determine participant adherence with the personal air monitoring protocol, motion detectors were installed in the backpacks of randomly selected participants. Across those participants, 95% of detected motion occurred during waking hours. This was consistent with verbal reports provided by all subjects that they were compliant with the protocol (i.e., wore the backpack during daytime hours over the monitoring period). For quality control, all personal monitoring results were coded for accuracy in flow rate, time, and completeness of documentation. Codes of 0-1 indicated high quality, 2 indicated intermediate quality, and 3 indicated unacceptable quality. Analyses were restricted to participants with high quality samples; participants with lower quality codes were excluded casewise.

Attrition Analyses.

Little’s Missing Completely at Random (MCAR) test assessed for patterns of missingness in the analytic sample. If patterns of missingness were revealed, we planned to examine differences in demographic characteristics between families who returned for the age 11 assessments versus those who did not. Specifically, we planned to conduct Wilcoxon rank sum tests and Pearson’s Chi-squared tests for categorical and continuous baseline characteristics, respectively.

Assessment of Covariates.

Maternal ethnoracial identification was determined by maternal self-report. For the purposes of the current analyses, ethnoracial identification was binarized into either Black or Latiné. Maternal nativity was also determined by maternal self-report of her country of birth. Maternal nativity was binarized into categories of United States born or born outside the United States. Maternal years of education were acquired by self-reported years of education after the first grade, obtained at the third trimester visit. Infants’ sex at birth and birth weight were determined by medical chart review at birth. Presence of a smoker in the home was self-reported by mothers during the third trimester visit. Home heat sources are known to increase indoor air pollution; as such, heating season is a derived variable to control for indoor air pollution confounding and is based on children’s date of birth (i.e., November to April; 3,4). Maternal intelligence was operationalized as mothers’ total score on the Test of Nonverbal Intelligence, third edition (TONI-3; 5). Quality of the home environment was assessed via the Early Childhood Home Inventory, an observational assessment conducted via a trained research worker (6). The Early Childhood Home Inventory consists of a 55-item checklist divided into 7 subscales (physical environment, learning materials, modeling, fostering self-sufficiency, regulatory activities, family companionship, and acceptance) and a total sum score; the total sum score was used in all analyses. The TONI-3 and the Home Environment Questionnaire were administered at children’s preschool visit (child mean age = 3.823, SD = 2.18). To account for potential changes in polycyclic aromatic hydrocarbon (PAH) exposure related to differences in the child’s built environment, parents reported whether they had moved at any point between the prenatal visit and the age 5 visit. Responses were binarized such that families were classified as either having moved or not moved.

**Supplementary Results**

Attrition Analyses.

Results revealed that missingness was not completely at random, as expected with longitudinal studies (χ^2^[77]=115.51, p=0.003; Table S4). There were 6 patterns of missingness in the data. Demographic comparison between participants who completed the age 11 visit and those that did not indicate mothers of children who did not return for the age 11 visit reported lower prenatal demoralization and were exposed to lower levels of PAHs during pregnancy than mothers of children who completed the age 11 visit (Table S5).

Table S1. Outlier Cases In Exposures and Outcomes

| Variable | N | Percent of Sample |
| --- | --- | --- |
| Demoralization | 2 | 0.38 |
| PAH | 4 | 0.75 |
| Age 7 Irritability | 5 | 1.03 |
| Age 9 Irritability | 7 | 1.54 |
| Age 11 Irritability | 3 | 0.82 |

Note: Outliers = |Z=±3|

Table S2. Comparison of demographic and baseline characteristics between included and excluded participants

|  | Included | Excluded | p-value |
| --- | --- | --- | --- |
|  | (N = 394) | (N = 333) |  |
| Demographic Variables |  |  |  |
| Sex at Birth |  |  | 0.375 |
| Sex (% Male) | 52.571 | 47.429 |  |
| Sex (% Female) | 55.851 | 44.149 |  |
| Smoker at Home |  |  | 0.73 |
| Smoker at Home (% yes) | 54.065 | 45.935 |  |
| Smoker at Home (% no) | 55.414 | 44.586 |  |
| Heat Season (% yes) | 57.79 | 42.21 | 0.143 |
| Maternal Nativity (% Native) |  |  | 0.223 |
| Born in the United States | 57.1 | 42.9 |  |
| Born Outside the United States | 52.296 | 47.704 |  |
| Maternal Age at Child Birth | 24.873 (4.904) | 24.480 (4.923) | 0.284 |
| Maternal years of education at prenatal visit (SD) | 11.888 (2.07) | 11.778 (2.312) | 0.5 |
| Maternal IQ (SD) | 85.094 (13.418) | 86.304 (12.939) | 0.301 |
| Child birthweight in grams (SD) | 3371.675 (482.467) | 3369.646 (451.520) | 0.955 |
| Moved |  |  | 0.346 |
| Moved (%yes) | 72.5 | 27.5 |  |
| Moved (%no) | 76.796 | 23.204 |  |
| Average Home Environment Score (SD)^a^ | 39.112 (6.227) | 40.144 (6.482) | 0.091 |
| Race |  |  | 0.013 |
| Latiné (%) | 50.74 | 49.26 |  |
| Black (%) | 60.63 | 39.37 |  |
| Baseline Measures |  |  |  |
| Prenatal PAH Exposure ^b^ | 0.834 (0.738) | 0.921 (0.772) | 0.133 |
| Maternal Demoralization | 0.412 (0.13) | 0.429 (0.135) | 0.0975 |

Note. ^a.^ Quality of the home environment measured by total score from HOME scale. ^b.^ Z-scaled natural logarithm of prenatal PAH exposure measured by personal air monitoring in the third trimester. N= number of participants; SD = standard deviation; PAH = polycyclic aromatic hydrocarbons; IQ = Intelligence Quotient

Table S3. Demographic characteristics of participants at each study visit

|  | Prenatal | Age 7 | Age 9 | Age 11 | Total |
| --- | --- | --- | --- | --- | --- |
| N | 395 | 391 | 361 | 285 | 394 |
| Mean child age in years (SD) | - | 6.52 (0.51) | 8.51 (0.51) | 10.56 (0.58) | 7.5 (2.34) |
| Sex (% male) | 262 (49.06) | 232 (47.84) | 213 (46.81) | 167 (45.88) | 272 (49.46) |
| Average Prenatal PAH Exposure (SD)^a^ | 0.84 (0.73) | 0.84 (0.72) | 0.87 (0.72) | 0.95 (0.70) | 0.84 (0.73) |
| Average Maternal Demoralization (SD) | 0.4167 (0.13) | 0.4130 (0.14) | 0.4173 (0.13) | 0.4207 (0.13) | 0.4157 (0.13) |
| Smoker at Home (% yes) | 177 (33.40) | 156 (32.43) | 147 (32.59) | 124 (34.54) | 185 (33.27) |
| Heat Season (% yes) | 264 (49.53) | 238 (48.97) | 224 (49.12) | 175 (48.08) | 275 (49.19) |
| Child birthweight in grams (SD) | 3373 (473.61) | 3390 (479.49) | 3376 (477.68) | 3385 (481.14) | 3370 (474.49) |
| Maternal Nativity (% Native) | 261 (51.12) | 235 (48.45) | 231 (50.77) | 182 (50.00) | 270 (48.21) |
| Maternal Age at Child Birth (SD) | 24.83 (4.92) | 24.91 (4.92) | 24.98 (4.96) | 24.91 (5.01) | 24.81 (4.91) |
| Maternal years of education at prenatal visit (SD) | 11.89 (2.16) | 11.86 (2.14) | 11.87 (2.13) | 11.94 (1.95) | 11.89 (2.16) |
| Maternal IQ (SD) | 85.10 (13.15) | 85.21 (13.27) | 84.78 (12.77) | 85.08 (12.99) | 85.09 (13.16) |
| Average Home Environment Score (SD)^b^ | 39.28 (6.28) | 39.13 (6.29) | 39.36 (6.03) | 39.53 (6.09) | 39.28 (6.37) |
| Moved (% yes) | 139 (29.51) | 145 (29.90) | 131 (29.37) | 104 (29.63) | 146 (29.67) |
| Latiné (%) | 333 (62.24) | 299 (61.52) | 276 (60.53) | 226 (62.08) | 352 (62.74) |
| Black (%) | 202 (37.76) | 187 (38.48) | 180 (39.47) | 138 (37.91) | 209 (37.25) |

Note. ^a.^ Z-scaled natural logarithm of prenatal PAH exposure measured by personal air monitoring in the third trimester. ^b.^ Quality of the home environment measured by total score from HOME scale. N= number of participants; SD = standard deviation; PAH = polycyclic aromatic hydrocarbons; IQ = Intelligence Quotient

| Table S4. Proportion of Missingness Across Analytic Variables | |
| --- | --- |
| Variable | Proportion Missing |
| Age 7 Irritability | 0.01 |
| Age 9 Irritability | 0.09 |
| Age 11 Irritability | 0.28 |
| Prenatal PAH Exposure | 0.00 |
| Maternal Demoralization | 0.00 |
| Interaction (PAH X MD) | 0.00 |
| Sex | 0.00 |
| Birth Weight | 0.00 |
| Race | 0.00 |
| Smoker at Home | 0.00 |
| Maternal IQ | 0.00 |
| Maternal Years of Education | 0.00 |
| Home Environment | 0.00 |
| Moved | 0.00 |
| DOB Heat Season | 0.00 |
| Maternal Nativity | 0.00 |
| Maternal Age at Child Birth | 0.00 |

| Table S5. Comparison of Demographic Differences in Sample with Returned for the Age 11 Visit and Those Who Did Not | | | |
| --- | --- | --- | --- |
| **Characteristic** | **Returned for Age 11 Visit**  N = 284^1^ | **Did Not Return**  N = 110^1^ | **p-value**^2^ |
| Prenatal PAH Exposure^a^ | 0.13 (0.93) | -0.41 (0.95) | <0.001 |
| Maternal Demoralization | 0.05 (0.97) | -0.23 (0.95) | 0.005 |
| Interaction (PAH X MD) | 0.03 (0.42) | -0.17 (0.37) | <0.001 |
| Birth Weight | -0.01 (1.04) | 0.05 (0.98) | 0.7 |
| Maternal IQ | 0.00 (0.99) | -0.01 (1.09) | 0.4 |
| Maternal Years of Education | -0.01 (0.89) | 0.01 (1.10) | >0.9 |
| Home Environment^b^ | 0.02 (0.93) | -0.13 (1.08) | 0.3 |
| Maternal Age at Child Birth | 0.01 (1.03) | 0.02 (0.92) | 0.7 |
| Sex | 127 (45%) | 57 (52%) | 0.2 |
| Race |  |  | 0.5 |
| Latinx | 170 (60%) | 70 (64%) |  |
| Black | 114 (40%) | 40 (36%) |  |
| Smoker at Home |  |  | 0.3 |
| No | 184 (65%) | 77 (70%) |  |
| Yes | 100 (35%) | 33 (30%) |  |
| Moved |  |  | 0.9 |
| Moved before age 7 | 83 (29%) | 33 (30%) |  |
| Did not Move | 201 (71%) | 77 (70%) |  |
| DOB Heat Season |  |  | 0.7 |
| Heating | 149 (52%) | 55 (50%) |  |
| Non-heating | 135 (48%) | 55 (50%) |  |
| Maternal Nativity | 143 (50%) | 46 (42%) | 0.13 |
| ^1^Mean (SD); n (%) | | | |
| ^2^Wilcoxon rank sum test; Pearson's Chi-squared test | | | |

| Table S6. Latent Growth Curve Model Results in Mothers with Highest Quartile Maternal Demoralization | | | | | | | | | | |
| --- | --- | --- | --- | --- | --- | --- | --- | --- | --- | --- |
| Variable | Intercept | | | | | Slope | | | | |
|  | B | Beta | SE | p-value | Confidence Interval | B | Beta | SE | p-value | Confidence Interval |
| Prenatal PAH Exposure^a^ | -1.120 | -1.265 | 0.779 | 0.151 | (−2.648, 0.407) | 0.412 | 3.474 | 0.218 | 0.059 | (−0.015, 0.840) |
| Maternal Demoralization | 0.067 | 0.049 | 0.164 | 0.681 | (−0.254, 0.389) | -0.061 | -0.333 | 0.047 | 0.193 | (−0.153, 0.031) |
| Interaction (PAH X MD) | 2.382 | 1.635 | 1.283 | 0.063 | (−0.133, 4.897) | -0.679 | -3.477 | 0.357 | 0.057 | (−1.379, 0.020) |
| Sex | 0.904 | 0.525 | 0.187 | 0.000 | (0.538, 1.270) | -0.085 | -0.367 | 0.055 | 0.124 | (−0.193, 0.023) |
| Birth Weight | -0.088 | -0.105 | 0.093 | 0.346 | (−0.270, 0.094) | -0.002 | -0.016 | 0.026 | 0.944 | (−0.053, 0.050) |
| Race | -0.120 | -0.132 | 0.131 | 0.360 | (−0.378, 0.137) | 0.009 | 0.071 | 0.037 | 0.813 | (−0.063, 0.081) |
| Smoker at Home | 0.146 | 0.178 | 0.095 | 0.125 | (−0.040, 0.333) | -0.023 | -0.212 | 0.027 | 0.392 | (−0.077, 0.030) |
| Maternal IQ | -0.205 | -0.249 | 0.109 | 0.059 | (−0.418, 0.008) | 0.059 | 0.532 | 0.030 | 0.048 | (0.000, 0.117) |
| Maternal Years of Education | 0.088 | 0.094 | 0.124 | 0.478 | (−0.156, 0.332) | -0.001 | -0.012 | 0.039 | 0.970 | (−0.078, 0.075) |
| Home Environment^b^ | 0.114 | 0.135 | 0.102 | 0.265 | (−0.086, 0.314) | -0.029 | -0.258 | 0.029 | 0.312 | (−0.086, 0.027) |
| Moved | -0.168 | -0.212 | 0.091 | 0.066 | (−0.347, 0.011) | 0.042 | 0.396 | 0.027 | 0.117 | (−0.011, 0.095) |
| DOB Heat Season | -0.137 | -0.081 | 0.202 | 0.497 | (−0.533, 0.259) | 0.116 | 0.515 | 0.056 | 0.039 | (0.006, 0.227) |
| Maternal Nativity | 0.051 | 0.029 | 0.283 | 0.858 | (−0.504, 0.605) | 0.101 | 0.435 | 0.078 | 0.195 | (−0.052, 0.255) |
| Maternal Age at Child Birth | -0.013 | -0.014 | 0.112 | 0.909 | (−0.232, 0.207) | 0.046 | 0.384 | 0.032 | 0.154 | (−0.017, 0.109) |
| Note: B= unstandardized coefficient; Beta = standardized coefficient; SE = standard error; ^a.^ Z-scaled natural logarithm of prenatal PAH exposure measured by personal air monitoring in the third trimester. ^b.^ Quality of the home environment measured by total score from HOME scale. N= number of participants; PAH = polycyclic aromatic hydrocarbons; MD = Maternal Demoralization; IQ = Intelligence Quotient; DOB = Date of birth | | | | | | | | | | |

| Table S7. Latent Growth Curve Model Results in Sample with Complete Case Irritability Data | | | | | | | | | | |
| --- | --- | --- | --- | --- | --- | --- | --- | --- | --- | --- |
| Variable | Intercept | | | | | Slope | | | | |
|  | B | Beta | SE | p-value | Confidence Interval | B | Beta* | SE | p-value | Confidence Interval |
| Prenatal PAH Exposure^a^ | -0.688 | -0.958 | 0.185 | 0.000 | (−1.050, −0.326) | 0.072 | - | 0.047 | 0.124 | (−0.020, 0.165) |
| Maternal Demoralization | 0.146 | 0.212 | 0.056 | 0.010 | (0.036, 0.257) | 0.011 | - | 0.014 | 0.426 | (−0.017, 0.039) |
| Interaction (PAH X MD) | 1.565 | 0.976 | 0.407 | 0.000 | (0.767, 2.363) | -0.148 | - | 0.104 | 0.154 | (−0.351, 0.055) |
| Sex | 0.310 | 0.230 | 0.104 | 0.003 | (0.106, 0.514) | -0.055 | - | 0.026 | 0.036 | (−0.107, −0.004) |
| Birth Weight | -0.021 | -0.032 | 0.051 | 0.684 | (−0.121, 0.080) | -0.002 | - | 0.013 | 0.857 | (−0.028, 0.023) |
| Race | 0.127 | 0.192 | 0.077 | 0.100 | (−0.024, 0.278) | 0.001 | - | 0.019 | 0.948 | (−0.037, 0.039) |
| Smoker at Home | 0.058 | 0.088 | 0.055 | 0.293 | (−0.050, 0.166) | -0.025 | - | 0.014 | 0.076 | (−0.052, 0.003) |
| Maternal IQ | -0.030 | -0.044 | 0.057 | 0.603 | (−0.141, 0.082) | 0.043 | - | 0.014 | 0.003 | (0.015, 0.071) |
| Maternal Years of Education | 0.074 | 0.098 | 0.067 | 0.268 | (−0.057, 0.205) | -0.002 | - | 0.017 | 0.900 | (−0.035, 0.031) |
| Home Environment^b^ | -0.088 | -0.122 | 0.061 | 0.150 | (−0.207, 0.032) | 0.006 | - | 0.015 | 0.698 | (−0.024, 0.036) |
| Moved | -0.054 | -0.080 | 0.053 | 0.305 | (−0.157, 0.049) | 0.014 | - | 0.013 | 0.303 | (−0.012, 0.040) |
| DOB Heat Season | 0.010 | 0.008 | 0.107 | 0.925 | (−0.200, 0.221) | 0.019 | - | 0.027 | 0.491 | (−0.035, 0.072) |
| Maternal Nativity | -0.262 | -0.196 | 0.161 | 0.104 | (−0.578, 0.054) | 0.051 | - | 0.041 | 0.214 | (−0.029, 0.130) |
| Maternal Age at Child Birth | -0.041 | -0.063 | 0.056 | 0.459 | (−0.151, 0.068) | 0.011 | - | 0.014 | 0.418 | (−0.016, 0.039) |
| Note: B= unstandardized coefficient; Beta = standardized coefficient; SE = standard error; *=standardized coefficients for slope predictors were unavailable due to minimal interindividual variability. ^a.^ Z-scaled natural logarithm of prenatal PAH exposure measured by personal air monitoring in the third trimester. ^b.^ Quality of the home environment measured by total score from HOME scale. N= number of participants; PAH = polycyclic aromatic hydrocarbons; MD = Maternal Demoralization; IQ = Intelligence Quotient; DOB = Date of birth | | | | | | | | | | |

| Table S8. Latent Growth Curve Model Results Using Only Items from the Child Behavior Checklist | | | | | | | | | | |
| --- | --- | --- | --- | --- | --- | --- | --- | --- | --- | --- |
| Variable | Intercept | | | | | Slope | | | | |
|  | B | Beta | SE | p-value | Confidence Interval | B | Beta* | SE | p-value | Confidence Interval |
| Prenatal PAH Exposure^a^ | -0.175 | -0.289 | 0.151 | 0.247 | (−0.470, 0.121) | -0.017 | - | 0.048 | 0.716 | (−0.111, 0.076) |
| Maternal Demoralization | 0.117 | 0.194 | 0.049 | 0.018 | (0.020, 0.213) | 0.003 | - | 0.015 | 0.845 | (−0.026, 0.032) |
| Interaction (PAH X MD) | 0.541 | 0.385 | 0.349 | 0.121 | (−0.143, 1.225) | 0.044 | - | 0.107 | 0.683 | (−0.166, 0.254) |
| Sex | 0.089 | 0.076 | 0.092 | 0.335 | (−0.091, 0.268) | -0.021 | - | 0.028 | 0.440 | (−0.076, 0.033) |
| Birth Weight | 0.025 | 0.043 | 0.045 | 0.584 | (−0.064, 0.114) | -0.016 | - | 0.014 | 0.240 | (−0.042, 0.011) |
| Race | 0.058 | 0.100 | 0.068 | 0.394 | (−0.075, 0.191) | -0.002 | - | 0.020 | 0.915 | (−0.042, 0.038) |
| Smoker at Home | 0.062 | 0.107 | 0.049 | 0.209 | (−0.035, 0.159) | -0.020 | - | 0.015 | 0.173 | (−0.049, 0.009) |
| Maternal IQ | -0.043 | -0.075 | 0.050 | 0.396 | (−0.142, 0.056) | 0.042 | - | 0.015 | 0.005 | (0.013, 0.072) |
| Maternal Years of Education | 0.112 | 0.182 | 0.055 | 0.041 | (0.005, 0.219) | -0.016 | - | 0.017 | 0.372 | (−0.050, 0.019) |
| Home Environment^b^ | -0.032 | -0.054 | 0.051 | 0.530 | (−0.132, 0.068) | -0.007 | - | 0.016 | 0.676 | (−0.038, 0.025) |
| Moved | -0.007 | -0.012 | 0.046 | 0.882 | (−0.097, 0.084) | 0.005 | - | 0.014 | 0.742 | (−0.023, 0.032) |
| DOB Heat Season | -0.064 | -0.054 | 0.096 | 0.505 | (−0.251, 0.124) | 0.031 | - | 0.029 | 0.272 | (−0.025, 0.087) |
| Maternal Nativity | -0.038 | -0.032 | 0.146 | 0.795 | (−0.324, 0.249) | 0.023 | - | 0.043 | 0.591 | (−0.061, 0.107) |
| Maternal Age at Child Birth | -0.052 | -0.088 | 0.051 | 0.316 | (−0.152, 0.049) | 0.021 | - | 0.015 | 0.168 | (−0.009, 0.050) |
| Note: B= unstandardized coefficient; Beta = standardized coefficient; SE = standard error; *=standardized coefficients for slope predictors were unavailable due to minimal interindividual variability. ^a.^ Z-scaled natural logarithm of prenatal PAH exposure measured by personal air monitoring in the third trimester. ^b.^ Quality of the home environment measured by total score from HOME scale. N= number of participants; PAH = polycyclic aromatic hydrocarbons; MD = Maternal Demoralization; IQ = Intelligence Quotient; DOB = Date of birth | | | | | | | | | | |

| Table S9. Latent Growth Curve Model Results Using Only Items from the Conners Parent Report Scale | | | | | | | | | | |
| --- | --- | --- | --- | --- | --- | --- | --- | --- | --- | --- |
| Variable | Intercept | | | | | Slope | | | | |
|  | B | Beta | SE | p-value | Confidence Interval | B | Beta | SE | p-value | Confidence Interval |
| Prenatal PAH Exposure^a^ | -0.475 | -0.660 | 0.154 | 0.002 | (−0.777, −0.174) | 0.075 | 0.579 | 0.048 | 0.117 | (−0.019, 0.170) |
| Maternal Demoralization | 0.167 | 0.234 | 0.050 | 0.001 | (0.070, 0.265) | 0.025 | 0.194 | 0.015 | 0.093 | (−0.004, 0.054) |
| Interaction (PAH X MD) | 1.102 | 0.657 | 0.353 | 0.002 | (0.410, 1.794) | -0.155 | -0.510 | 0.107 | 0.148 | (−0.364, 0.055) |
| Sex | 0.345 | 0.247 | 0.095 | 0.000 | (0.158, 0.531) | -0.043 | -0.171 | 0.028 | 0.129 | (−0.099, 0.013) |
| Birth Weight | 0.008 | 0.012 | 0.047 | 0.863 | (−0.083, 0.099) | -0.003 | -0.021 | 0.014 | 0.854 | (−0.030, 0.024) |
| Race | -0.040 | -0.058 | 0.069 | 0.560 | (−0.175, 0.095) | 0.035 | 0.283 | 0.020 | 0.082 | (−0.005, 0.075) |
| Smoker at Home | -0.036 | -0.052 | 0.051 | 0.479 | (−0.137, 0.064) | -0.016 | -0.129 | 0.015 | 0.288 | (−0.046, 0.014) |
| Maternal IQ | 0.008 | 0.012 | 0.053 | 0.874 | (−0.096, 0.113) | 0.025 | 0.200 | 0.016 | 0.126 | (−0.007, 0.056) |
| Maternal Years of Education | 0.044 | 0.060 | 0.056 | 0.426 | (−0.065, 0.153) | 0.010 | 0.078 | 0.017 | 0.549 | (−0.024, 0.044) |
| Home Environment^b^ | -0.067 | -0.094 | 0.055 | 0.221 | (−0.174, 0.040) | 0.028 | 0.216 | 0.016 | 0.087 | (−0.004, 0.060) |
| Moved | 0.019 | 0.027 | 0.047 | 0.688 | (−0.074, 0.112) | 0.001 | 0.005 | 0.014 | 0.965 | (−0.027, 0.029) |
| DOB Heat Season | -0.102 | -0.073 | 0.099 | 0.306 | (−0.297, 0.093) | 0.026 | 0.104 | 0.029 | 0.372 | (−0.031, 0.084) |
| Maternal Nativity | 0.006 | 0.004 | 0.145 | 0.967 | (−0.279, 0.291) | 0.014 | 0.057 | 0.042 | 0.735 | (−0.069, 0.098) |
| Maternal Age at Child Birth | 0.018 | 0.026 | 0.052 | 0.725 | (−0.083, 0.120) | -0.011 | -0.084 | 0.015 | 0.480 | (−0.040, 0.019) |
| Note: B= unstandardized coefficient; Beta = standardized coefficient; SE = standard error; ^a.^ Z-scaled natural logarithm of prenatal PAH exposure measured by personal air monitoring in the third trimester. ^b.^ Quality of the home environment measured by total score from HOME scale. N= number of participants; PAH = polycyclic aromatic hydrocarbons; MD = Maternal Demoralization; IQ = Intelligence Quotient; DOB = Date of birth | | | | | | | | | | |
| Table S10. Latent Growth Curve Model Results with Outlier Data Removed | | | | | | | | | | |
| Variable | Intercept | | | | | Slope | | | | |
|  | B | Beta | SE | p-value | Confidence Interval | B | Beta* | SE | p-value | Confidence Interval |
| Prenatal PAH Exposure^a^ | 0.024 | 0.037 | 0.049 | 0.631 | (−0.073, 0.120) | 0.008 | - | 0.008 | 0.008 | (−0.020, 0.036) |
| Maternal Demoralization | 0.159 | 0.253 | 0.048 | 0.001 | (0.064, 0.253) | 0.005 | - | 0.005 | 0.005 | (−0.022, 0.032) |
| Interaction (PAH X MD) | 0.132 | 0.206 | 0.048 | 0.006 | (0.038, 0.226) | -0.014 | - | -0.014 | -0.014 | (−0.041, 0.013) |
| Sex | 0.219 | 0.183 | 0.088 | 0.013 | (0.046, 0.391) | -0.047 | - | -0.047 | -0.047 | (−0.096, 0.002) |
| Birth Weight | 0.014 | 0.023 | 0.043 | 0.753 | (−0.071, 0.099) | -0.005 | - | -0.005 | -0.005 | (−0.029, 0.019) |
| Race | 0.024 | 0.041 | 0.065 | 0.712 | (−0.104, 0.152) | 0.008 | - | 0.008 | 0.008 | (−0.028, 0.045) |
| Smoker at Home | 0.039 | 0.066 | 0.047 | 0.405 | (−0.053, 0.132) | -0.025 | - | -0.025 | -0.025 | (−0.051, 0.001) |
| Maternal IQ | -0.024 | -0.042 | 0.048 | 0.614 | (−0.119, 0.071) | 0.039 | - | 0.039 | 0.039 | (0.012, 0.066) |
| Maternal Years of Education | 0.071 | 0.113 | 0.052 | 0.175 | (−0.032, 0.173) | -0.011 | - | -0.011 | -0.011 | (−0.041, 0.020) |
| Home Environment^b^ | -0.062 | -0.102 | 0.049 | 0.205 | (−0.157, 0.034) | 0.009 | - | 0.009 | 0.009 | (−0.019, 0.037) |
| Moved | -0.018 | -0.030 | 0.044 | 0.688 | (−0.104, 0.068) | 0.014 | - | 0.014 | 0.014 | (−0.011, 0.038) |
| DOB Heat Season | -0.052 | -0.044 | 0.092 | 0.568 | (−0.233, 0.128) | 0.009 | - | 0.009 | 0.009 | (−0.042, 0.059) |
| Maternal Nativity | 0.008 | 0.007 | 0.139 | 0.956 | (−0.265, 0.281) | 0.025 | - | 0.025 | 0.025 | (−0.051, 0.101) |
| Maternal Age at Child Birth | -0.018 | -0.030 | 0.049 | 0.712 | (−0.114, 0.078) | 0.010 | - | 0.010 | 0.010 | (−0.017, 0.036) |
| Note: B= unstandardized coefficient; Beta = standardized coefficient; SE = standard error; *=standardized coefficients for slope predictors were unavailable due to minimal interindividual variability. ^a.^ Z-scaled natural logarithm of prenatal PAH exposure measured by personal air monitoring in the third trimester. ^b.^ Quality of the home environment measured by total score from HOME scale. N= number of participants; PAH = polycyclic aromatic hydrocarbons; MD = Maternal Demoralization; IQ = Intelligence Quotient; DOB = Date of birth | | | | | | | | | | |


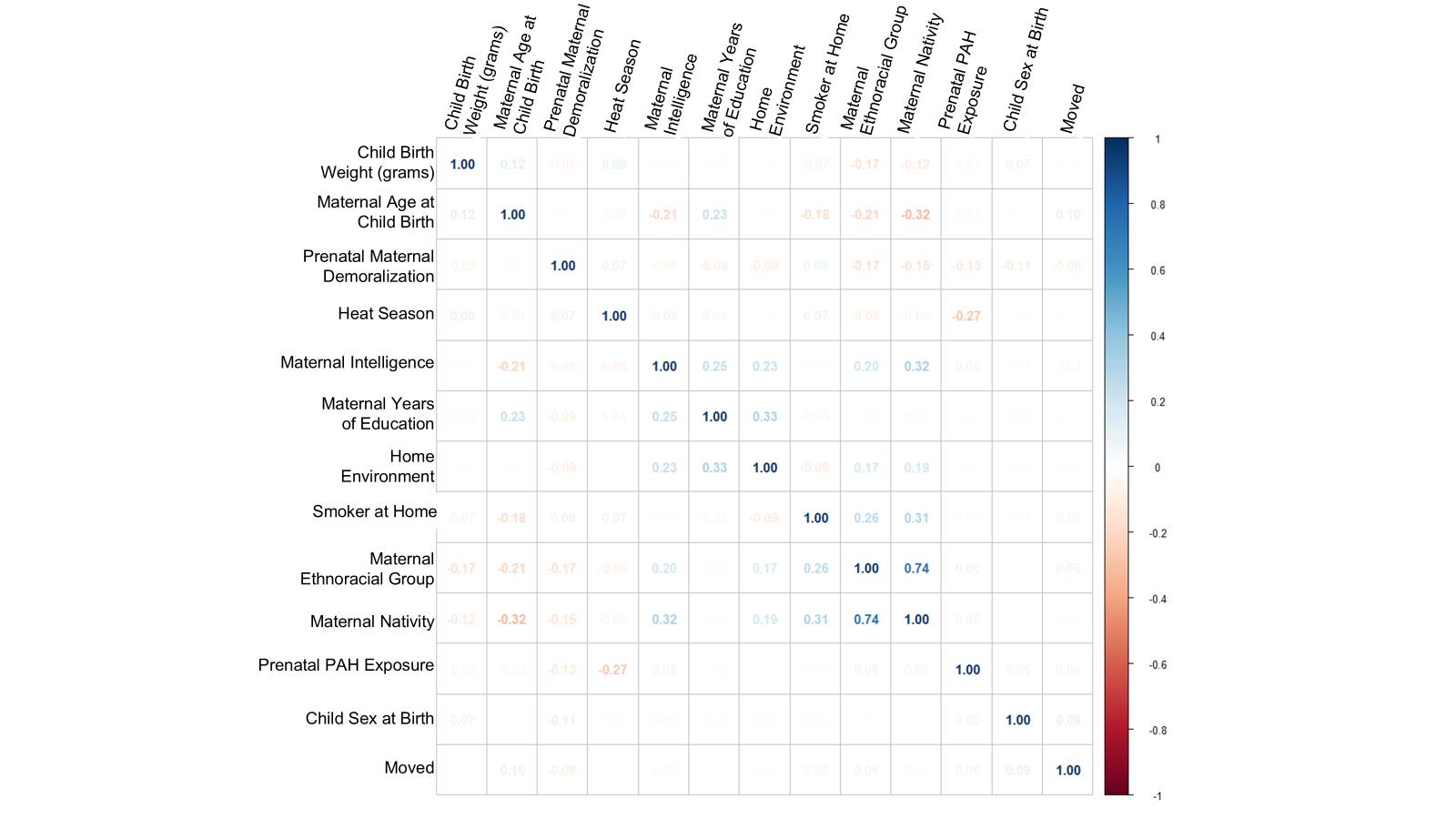


Figure S1. Intercorrelations between untransformed confounding variables. Scale color denotes strength of correlations, blue = positive, red = negative.

Supplementary References

1. Perera FP, Rauh V, Tsai W-Y, Kinney P, Camann D, Barr D, Bernert T, Garfinkel R, Tu Y-H, Diaz D, et al. Effects of transplacental exposure to environmental pollutants on birth outcomes in a multiethnic population. *Environ Health Perspect* (2003) 111:201–205.

2. Perera FP, Illman SM, Kinney PL, Whyatt RM, Kelvin EA, Shepard P, Evans D, Fullilove M, Ford J, Miller RL, et al. The challenge of preventing environmentally related disease in young children: community-based research in New York City. *Environ Health Perspect* (2002) 110:197–204.

3. Jung KH, Patel MM, Moors K, Kinney PL, Chillrud SN, Whyatt R, Hoepner L, Garfinkel R, Yan B, Ross J, et al. Effects of Heating Season on Residential Indoor and Outdoor Polycyclic Aromatic Hydrocarbons, Black Carbon, and Particulate Matter in an Urban Birth Cohort. *Atmos Environ*  (2010) 44:4545–4552.

4. Gould CF, Chillrud SN, Phillips D, Perzanowski MS, Hernández D. Soot and the city: Evaluating the impacts of Clean Heat policies on indoor/outdoor air quality in New York City apartments. *PLoS One* (2018) 13:e0199783.

5. Brown L, Sherbenou RJ, Johnson SK. *Test of Nonverbal Intelligence*. third. Austin, TX: Pro-Ed. (1997).

6. Caldwell, B. M., & Bradley, R. H. *Home Observation for Measurement of the Environment: Administration Manual*. Tempe, AZ: Family & Human Dynamics Research Institute, Arizona State University. (2003).
